# Cardiac Mass and Cerebrovascular Accident as Primary Manifestations of Churg-Strauss Syndrome

**DOI:** 10.34172/aim.2023.97

**Published:** 2023-11-01

**Authors:** Fariba Abbasi, Ata Abbasi, Alireza Rostamzadeh, Seyede Zohre Banihashemi, Aliakbar Rajabi

**Affiliations:** ^1^Solid Tumor Research Center, Cellular and Molecular Medicine Research Institute, Urmia University of Medical Sciences, Urmia, Iran; ^2^Department of Pathology, Faculty of Medicine, Urmia University of Medicine, Urmia, Iran; ^3^Department of Cardiology, Faculty of Medicine, Urmia University of Medical Sciences, Urmia, Iran

**Keywords:** Cardiac mass, Churg-Strauss syndrome, Eosinophilia, Granuloma, Vasculitis

## Abstract

Churg-Strauss syndrome (CSS), recently named eosinophilic granulomatosis with polyangiitis (EGPA), is a rare form of systemic vasculitis with extravascular granulomas occurring in patients with asthma and tissue eosinophilia. We represent a large left ventricular granuloma, confirmed by histopathologic evaluation, detected as a ventricular mass by echocardiography in a 45-year-old asthmatic male who was admitted for a cerebrovascular accident. Paraclinical and histopathologic findings confirmed the diagnosis of EGPA. As cardiac involvement in patients with EGPA is associated with poor prognosis, routine echocardiographic evaluation of these patients is suggested.

## Introduction

 Churg-Strauss syndrome (CSS), recently named eosinophilic granulomatosis with polyangiitis (EGPA), is a rare form of necrotizing vasculitis involving small and medium-sized arteries.^1-4^ The major clinical manifestations are asthma, sinusitis, transient pulmonary infiltrate and neurologic manifestations commonly as peripheral neurologic symptoms and less commonly in the form of central nervous system involvement.^3,5,6^ Due to positive anti-neutrophil cytoplasmic antibodies (ANCA) in 40%‒60% of cases, this syndrome is classified as one of the ANCA-associated vasculitides.^3,4,7^ Histopathology examination mostly reveals extravascular granulomas, tissue eosinophilia and necrotizing vasculitis.^8^

 Cardiac involvement is seen in 15%‒60% of the patients, especially in ANCA-negative cases.^3,4,9^ The manifestations are variable, including coronary vasculitis, constructive pericarditis, pericardial effusion, myocarditis, myocardial infarction and fibrosis.^1,3,10^ Generally, cardiac involvement is associated with worse outcome, accounting for approximately one-half of disease-related deaths.^2,3,8,9,11^

 Here, we report a case of CSS admitted to the hospital with a cerebrovascular accident and cardiac involvement presenting as a ventricular mass which is a very rare form of cardiac involvement in this syndrome.

## Case Report

 The patient was a 45-year-old man with a 3-year history of sinusitis, nonproductive cough, exertional dyspnea and malaise who was under treatment for allergic asthma with symbicort, rhinocort and desloratadine.

 Two months earlier, he was brought to the hospital with sudden severe headache, speech disorder and right-sided hemiparesis. Brain computed tomography (CT) scan and magnetic resonance imaging (MRI) findings were consistent with cerebrovascular accident.

**Figure 1:**
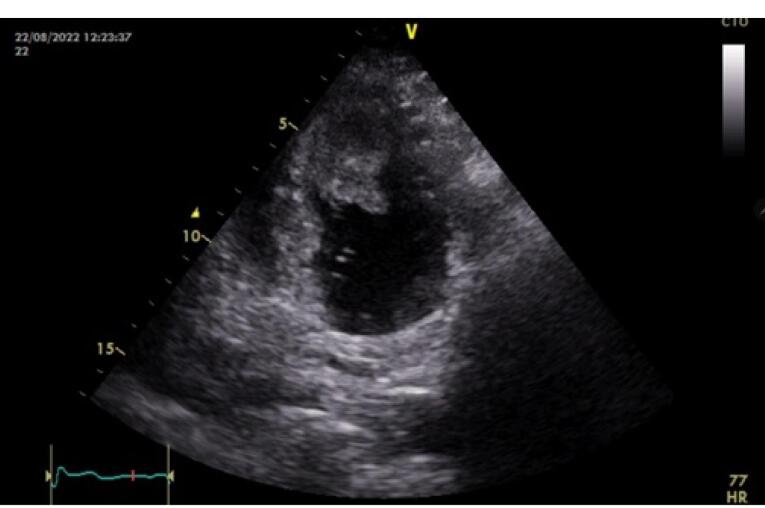
Echocardiography of the Left Ventricular Mass

 Echocardiography was performed during hospitalization and showed a left ventricular echogenic semi-mobile mass measuring 5.2 × 4.5 × 1.2 cm with multiple echolucency and strand like projections which had filled the apex (Figure 1).

 The laboratory findings were as follow: ESR: 62/h, CRP: 2 + , WBC: 9100/mL with 11.6% eosinophilia. ANA, anti dsDNA, P-ANCA and C-ANCA were negative. Other tests were in normal ranges.

**Figure 2:**
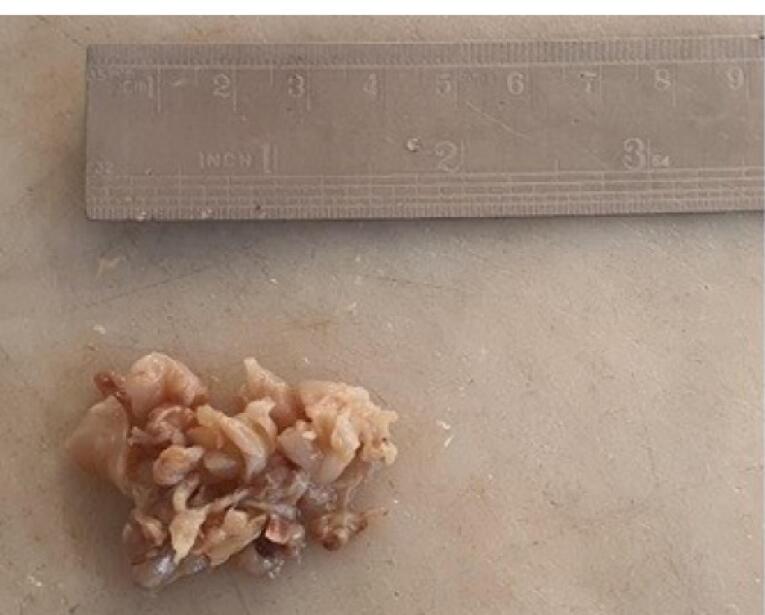
Gross Appearance of the Mass; Multiple Pieces of Creamy Colored Tissue

**Figure 3:**
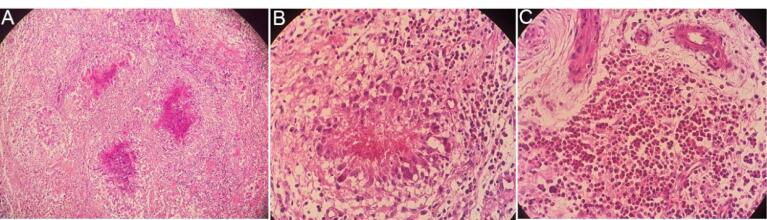
Microscopic Findings of the Mass; **(A)** Multiple granulomas (x100). **(B)** Granuloma with fibrinoid necrosis (x400). **(C)** Severe eosinophilic infiltration (x400)

 The patient underwent surgery. The specimen received ed in the pathology department consisted of multiple pieces of creamy colored tissue, rubbery in consistency measuring 4 × 3 × 2 cm in aggregate (Figure 2). Microscopic examination showed fibrotic tissue with many thick-walled vessels, severe infiltration of inflammatory cells composed predominantly of eosinophils, many granulomas with central fibrinoid necrosis or basophilic appearance with palisading of histiocytes and giant cells, cell debris and extensive areas of fibrin deposition (Figure 3).

 Currently, according to the American College of Rheumatology, there are six diagnostic criteria for diagnosis of CSS, namely: (1) History of wheezing or diffuse high-pitched expiratory rhonchi, (2) Eosinophilia, (3) Mono- or polyneuropathy, (4) Pulmonary infiltrates attributable to vasculitis, (5) History of paranasal sinus abnormality or pain, and (6) Extravascular eosinophils confirmed by biopsy.^12^ The main differential diagnoses of CCS are granulomatosis with polyangiitis and microscopic polyangiitis.

 Our patient had 4 items of the mentioned criteria including history of wheezing and asthma, eosinophilia, history of sinusitis and a biopsy confirming the presence of eosinophilic granuloma.

 The combination of laboratory findings and histologic features confirmed the diagnosis of EGPA.

## Discussion

 CSS is a rare systemic disorder; cardiac involvement is seen in 15%‒60% of the cases.^3,4,9^ Active asthma or a history of asthma is seen in more than 90% of patients with CSS.^8^ Our patient also had a 3-year history of asthma. Atopy in the form of sinusitis or allergic rhinitis, cough and hemoptysis, arthralgia, gastrointestinal tract involvement and skin changes are frequent in these patients.^8^ Our patient had nonproductive cough with sinusitis and rhinitis but no hemoptysis, arthralgia or skin involvement. Peripheral neurologic symptoms are seen in 55%‒75% of the patients but involvement of the central nervous system is less common.^5^ The presented patient had a history of cerebrovascular accident before the establishment of the diagnosis.

 Cardiac involvement in CSS is mostly seen in ANCA-negative patients, as was the case for our patient.^3,4^ The dominant histopathologic features of the involved heart are eosinophilic infiltration and rarely necrotizing vasculitis or extravascular granuloma.^9,11^ Eosinophilic infiltration and multiple granulomas with basophilic appearance and fibrinoid necrosis, fibrosis and fibrin deposition are the histologic features observed in our samples.

 After confirmation of the diagnosis of EGPA, the patient underwent treatment with prednisolone, montelukast, fexofenadine and hydroxychloroquine. One week later, eosinophilia decreased to 8.2%.

 In conclusion,as cardiac involvement in CSS is associated with a poor outcome, early diagnosis of this complication is very important. This clinical presentation demonstrated the importance of echocardiography as a reliable non-invasive technique in the assessment of cardiac involvement in CSS. Also, CSS should be considered in differential diagnosis of cardiac masses in patient with blood eosinophilia.
